# DNA N^6^-methyladenine demethylase ALKBH1 enhances osteogenic differentiation of human MSCs

**DOI:** 10.1038/boneres.2016.33

**Published:** 2016-10-11

**Authors:** Chenchen Zhou, Yuting Liu, Xiaobing Li, Jing Zou, Shujuan Zou

**Affiliations:** 1 State Key Laboratory of Oral Diseases, West China Hospital of Stomatology, Sichuan University, Chengdu, China

## Abstract

ALKBH1 was recently discovered as a demethylase for DNA N^6^-methyladenine (N6-mA), a new epigenetic modification, and interacts with the core transcriptional pluripotency network of embryonic stem cells. However, the role of ALKBH1 and DNA N6-mA in regulating osteogenic differentiation is largely unknown. In this study, we demonstrated that the expression of ALKBH1 in human mesenchymal stem cells (MSCs) was upregulated during osteogenic induction. Knockdown of ALKBH1 increased the genomic DNA N6-mA levels and significantly reduced the expression of osteogenic-related genes, alkaline phosphatase activity, and mineralization. ALKBH1-depleted MSCs also exhibited a restricted capacity for bone formation *in vivo*. By contrast, the ectopic overexpression of ALKBH1 enhanced osteoblastic differentiation. Mechanically, we found that the depletion of ALKBH1 resulted in the accumulation of N6-mA on the promoter region of *ATF4*, which subsequently silenced *ATF4* transcription. In addition, restoring the expression of *ATP* by adenovirus-mediated transduction successfully rescued osteogenic differentiation. Taken together, our results demonstrate that ALKBH1 is indispensable for the osteogenic differentiation of MSCs and indicate that DNA N6-mA modifications area new mechanism for the epigenetic regulation of stem cell differentiation.

## Introduction

Stem cells are characterized by two features: the ability to differentiate into multiple cell types and the ability to self-renew.^1,2^ Mesenchymal stem cells (MSCs) are one type of postnatal stem cell with a pluripotent differentiation potential that is broader than originally envisioned or perhaps as broad as that of embryonic stem cells.^2^ MSCs have the ability to differentiate into different mesenchymal lineages, such as osteoblasts, chondrocytes, adipocytes, fibroblasts, and adventitial reticular cells.^3^ Consequently, MSCs can be seen as bona fide cells for all tissues in which they induce osteoprogenitors and then transform into osteoblasts,which are crucial for the mineralization of the extracellular matrix (ECM) of bone.^4–6^

The osteogenic differentiation of MSCs is regulated by multiple mechanisms, such as key transcription factors, including runt-related transcription factor 2 and Osterix,^2,5,7^ as well as other hormones.^1,8–10^ In addition, epigenetic regulations have an important role in mammalian biology^11,12^ and regulate tissue-specific gene expression.^13,14^ Recently, DNA methylation, which is an epigenetic regulation, was found to have a pivotal role in stem cell differentiation.^15^ DNA methylation occurs on the fifth position of cytosine (5mC).^16^ DNA cytosines experience a series of modifications performed by a variety of enzymes, including DNA methyltransferases,^17^ which add a methyl group on the fifth position of cytosine to form 5mC; TET family dioxygenases (TET1, TET2, and TET3),^18,19^ which then oxidize the methyl group to create 5-hydroxymethylcytosine;^20^ and 5-formylcytosine and 5-carboxylcytosine, which complete the cycle.^21^ The epigenetic activation of bone-specific genes mediated by promoter demethylation typically occurs when MSCs differentiate into osteoblasts,^22^ and the inhibition of stem-cell-specific genes by promoter methylation is a crucial epigenetic mechanism during stem cell differentiation.^23^

Very recently, the methylation of N^6^-methyladenine (N6-mA) has been reported as another DNA methylation event, and ALKBH1 was discovered as a demethylase for DNA N6-mA.^11,24^ ALKBH1, a member of the AlkB family, is a 2-oxoglutarate and Fe^2+^-dependent hydroxylase.^25,26^ ALKBH1 has an important role in epigenetic regulation by accommodating the expression of pluripotency markers and genes related to neural differentiation during embryogenesis.^27^ ALKBH1 is involved in fine-tuning the level of a core transcriptional network and regulating the developmental regulatory microRNAs involved in pluripotency and differentiation.^21^ Most of the *Alkbh1* ^*−/−*^mice died during embryogenesis, and survivors exhibit tissue developmental defects, including prolonging the expression of pluripotency markers,^28^ and multiple defects in eyes, craniofacial, sternum, and limb skeleton,^26^ which suggests that ALKBH1 is indispensable for stem differentiation and development. However, the role of ALKBH1 and DNA N6-mA in regulating osteogenic differentiation is largely unknown.

In this study, we demonstrated that the depletion or overexpression of ALKBH1 in human MSCs regulates the levels of genomic DNA N6-mA and significantly affects osteogenic differentiation and bone formation. Mechanically, we found that the depletion of ALKBH1 results in the accumulation of N6-mA on the promoter region of activating transcription factor 4 (ATF4), which subsequently silences *ATF4* transcription.

## Materials and methods

### Cell culture

Human bone marrow-derived MSCs were obtained from American Type Culture Collection (ATCC, Manassas, VA, USA). Cells were cultured in Dulbecco’s modified Eagle’s medium (DMEM) supplemented with 10% fetal bovine serum (Gibco, Carlsbad, CA, USA) plus 100 U·mL^−1^ of penicillin and 100 mg·mL^−1^ of streptomycin (Gibco) at 37 °C with a humidified atmosphere of 5% CO_2_. To induce osteogenic differentiation, MSCs were seeded in 6- or 24-well plates. After confluence, cells were treated with osteogenic medium containing 50 μmol·L^−1^ ascorbic acid, 10 mmol·L^−1^ β-glycerophosphate, and 10 nmol·L^−1^ dexamethasone (Sigma, Shanghai, China). All experimental protocols and procedures were approved by the State Key Laboratory of Oral Diseases, West China Hospital of Stomatology, Sichuan University.

### Gene knockdown and overexpression

ALKBH1-targeted and control small interfere RNAs were purchased from Santa Cruz (Dallas, TX, USA). Transfection was performed using Lipofectamine RNAiMAX reagent (Invitrogen) according to the manufacturer's instructions. Knockdown efficiency was determined by reverse transcription-PCR (RT-PCR) and western blot 2 days after the transfection. The lentivirus particles of ALKBH1 and scrambled shRNAs were obtained from Genecopoeia (Guangzhou, China). The stable cell lines were established by puromycin selection.

For ALKBH1 overexpression, lentiviruses expressing the human ALKBH1 gene were purchased from Genecopoeia. MSCs were infected with ALKBH1 or empty vectors in the presence of polybrene (Sigma) for 24 h and were selected with puromycin (Sigma). For ATF4 overexpression, the adenovirus particles expressing human ATF4 or GFP (control) were obtained from Cyagen (Guangzhou, China).

### RNA isolation and RT-PCR

Total RNA was isolated using the Trizol reagent (Invitrogen) according to the manufacturer’s instructions. The complementary DNA was prepared from 2 μg aliquots of RNA using a QuantiTec reverse transcription kit (Qiagen, Valencia, CA, USA).^29–31^ Quantitative real-time PCR was performed using SYBR Premix Ex Taq (Takara, Dalian, China) in an ABI7500 real-time PCR system (Applied Biosystems, Foster City, CA, USA). The primer sequences used are listed in Table 1. Relative expression was calculated using a 2^−ΔΔCt^ method^32^ by normalization with *Gapdh* housekeeping gene expression and presented as fold increase relative to control.

### Western blot

Cells were lysed in RIPA buffer (Pierce, Rockford, IL, USA) supplemented with a protease inhibitor cocktail (Roche, Mannheim, Germany) and centrifuged at 18 000 *g* for 15 min at 4 °C. The supernatants were heated at 95 °C for 5 min in sample buffer containing 2% SDS and 1% 2-mercaptoethanol, separated on 10% SDS–polyacrylamide gels, and transferred to polyvinylidene difluoride membranes using a semi-dry transfer apparatus (Bio-Rad).^33^ The membranes were blocked with 5% milk for 1 h and then incubated with anti-ALKBH1 (Millipore, Billerica, MA, USA, 1:1 000), anti-ATF4 (Abcam, Cambridge, MA, USA, 1:1 000) or anti-*α*-Tubulin (Sigma, 1:5 000) overnight followed by a horseradish peroxidase-conjugated anti-rabbit or anti-mouse IgG (Jackson ImmunoResearch, West Grove, PA, USA). The antibody–antigen complexes were visualized with SuperSignal reagents (Pierce, Rockford, IL, USA).

### Dot blot

Genomic DNA was isolated using a PureLink Genomic DNA kit (Invitrogen) and then denatured at 95 °C for 10 min in 0.4 mol·L^−1^ NaOH and 10 mmol·L^−1^ EDTA buffer. Samples were spotted on the membrane (Zeta-Probe, Bio-Rad, Hercules, CA, USA) using a Dot-Blot microfiltration apparatus (Bio-Rad) and baked at 80 °C for 30 min. Membranes were blocked in blocking buffer (5% milk in PBST) for 1 h at room temperature and incubated with N6-mA antibody (202-003, Synaptic Systems, Goettingen, Germany, 1:2 000) overnight at 4 °C. After three washes, membranes were incubated with horseradish peroxidase-linked secondary anti-rabbit IgG (Jackson ImmunoResearch). The antibody–antigen complexes were visualized with SuperSignal reagents (Pierce). To ensure an equal amount of DNA was spotted, the same membrane was stained with 0.02% methylene blue in 0.3 mol·L^−1^ sodium acetate (pH 5.2).

### ALP and Alizarin red staining

For alkaline phosphatase (ALP) staining, cells were grown in osteogenic differentiation medium for 7 days. Cells were then fixed in 70% ethanol and incubated with a staining solution of 0.25% naphthol AS-BI phosphate and 0.75% Fast Blue BB dissolved in 0.1 mol·L^−1^ Tris buffer (pH 9.3). We also quantified the ALP activity using a commercial kit according to the manufacturer’s protocol (Cell Biolab, San Diego, CA, USA).

For mineralization assays, cells were cultured in differentiation medium for 2–3 weeks, fixed with 70% ethanol, and stained with 40 mmol·L^−1^ Alizarin red S (pH 4.2, Sigma) for 10 min.^34^ Mineralized bone nodules stained with alizarin red were distained with 10% cetylpyridinium chloride in 10 mmol·L^−1^ sodium phosphate (pH 7.0), and the calcium concentration was determined by absorbance measurements at 562 nm.

### Ectopic bone formation

Three-month-old immunocompromised beige mice were obtained from the Experimental Animal Center of the University and housed in pathogen-free facilities under a 12-h light and 12-h dark cycle. All procedures were conducted in accordance with *The Guidelines for the Care and Use* of Laboratory Animals of State Key Laboratory of Oral Diseases, West China Hospital of Stomatology, Sichuan University. Approximately 5×10^6^ of cells were mixed with 60 mg of pure phase *β*-tricalcium phosphate particles (SynthoGraft, Bicon, Boston, MA, USA) and then transplanted subcutaneously under the dorsal surface as described previously.^11,24^ Six weeks after transplantation, the transplants were collected, fixed with 10% formalin, and decalcified with 10% EDTA. Paraffin sections were fabricated and stained with hematoxylin and eosin.^35^

### Chromatin immunoprecipitation assay

The chromatin immunoprecipitation assay was performed using a Simple ChIP Assay kit (Cell Signaling Technology, Danvers, MA, USA) according to the manufacturer’s protocol^31^ with an antibody against N6-mA (cat# 202003, Synaptic Systems) or the control normal rabbit IgG (cat#sc-2027, Santa Cruz). After dissociating the DNA–protein complexes, pulled down DNA along with the input DNA (devoid of antibody) were subjected to quantitative PCR analysis with primers to interrogate the ATF4 promoter (Table 1). The results are expressed as the percentage of input DNA.

### Statistical analysis

All values were presented as the mean±s.e. Two-tailed Student’s *t*-test and one-way analysis of variance followed by the Tukey’s test were used for single and multiple comparisons with assess the statistical inference on difference among each pair of data sets, respectively. A *P* value<0.05 was considered statistically significant.

## Results

### ALKBH1 is upregulated during osteogenic differentiation

**Figure 1:**
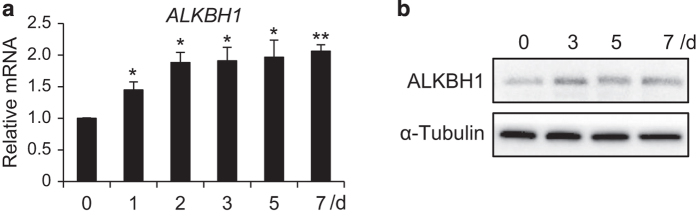
ALKBH1 is upregulated during osteogenic differentiation. (**a**) Real-time RT-PCR of *ALKBH1.n*=3. *P<0.05 and **P<0.01. (**b**) Western blot analysis.

We first evaluated the expression profile of ALKBH1 in human MSCs during osteogenic differentiation. As determined by real-time RT-PCR, the ALKBH1 messenger RNA levels were significantly upregulated in response to osteogenic induction (Figure 1a). This observation was also confirmed by western blot analysis (Figure 1b). These results suggest that ALKBH1 may have a role in the osteogenic differentiation of MSCs.

### Depletion of ALKBH1 inhibits osteogenic differentiation *in vitro*

**Figure 2:**
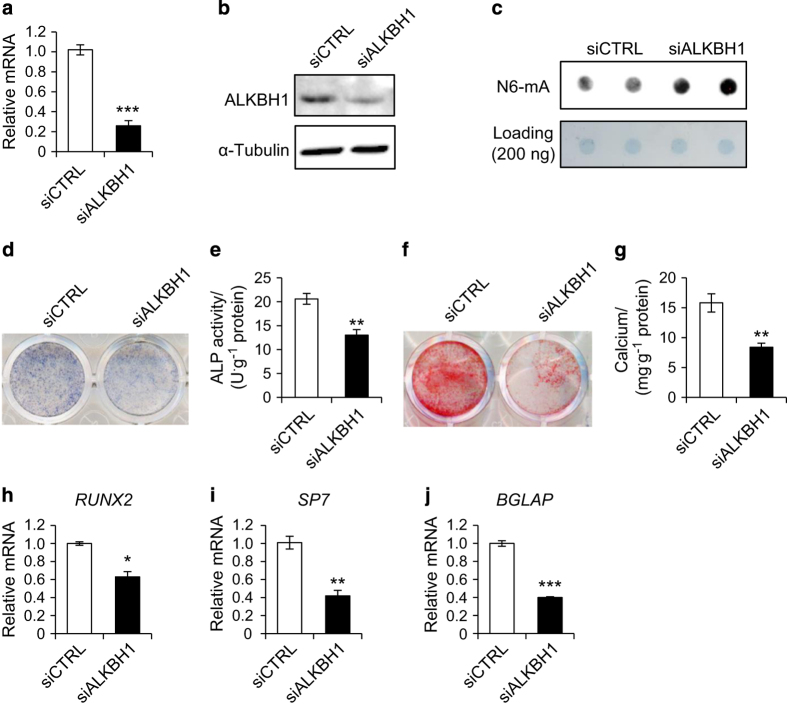
Depletion of ALKBH1 inhibits osteogenic differentiation. (**a**) Real-time RT-PCR*. n*=3. ***P<0.001. (**b**) Western blot analysis. (**c**) Representative images of dot blot assay. (**d**) Representative image of alkaline phosphatase (ALP) staining. (**e**) Quantitative analyses of the ALP activity. *n*=5. **P<0.01. (**f**) Representative image of Alizarin red S (ARS) staining of MSCs. (**g**) Quantitative analyses of calcium mineralization. *n*=5. **P<0.01. (**h**–**j**) Real-time RT-PCR revealed reduced *RUNX2*, *SP7*, and *BGLAP* messenger RNA expression. *n*=3. *P<0.05, **P<0.01 and ***P<0.001.

To investigate the role of ALKBH1 in osteogenic differentiation, we knocked down ALKBH1 in human MSCs. The knockdown efficiency was confirmed by RT-PCR and western blot (Figure 2a and b). Given that ALKBH1 was recently discovered as a demethylase for DNA N6-mA, we evaluated the modification of N6-mA using a DNA dot blot assay. As shown in Figure 2c, depletion of ALKBH1 markedly increased N6-mA levels in whole genomic DNA of MSCs.

After osteogenic induction for 7 days, we found that the small interfere RNA-mediated depletion of ALKBH1 significantly reduced ALP activity, which is an early marker of osteoblastic differentiation (Figure 2d and e). We also assessed ECM mineralization by Alizarin red S staining. As shown in Figure 2f and g, the mineralization was significantly decreased after ALKBH1 depletion. In addition, the knockdown of ALKBH1 inhibited the expression of osteogenic-related genes, such as *RUNX2*, *Osterix* (*SP7*), and *Osteocalcin* (*GBLAP*) (Figure 2h–j).

### Depletion of ALKBH1 inhibits bone formation *in vivo*

**Figure 3:**
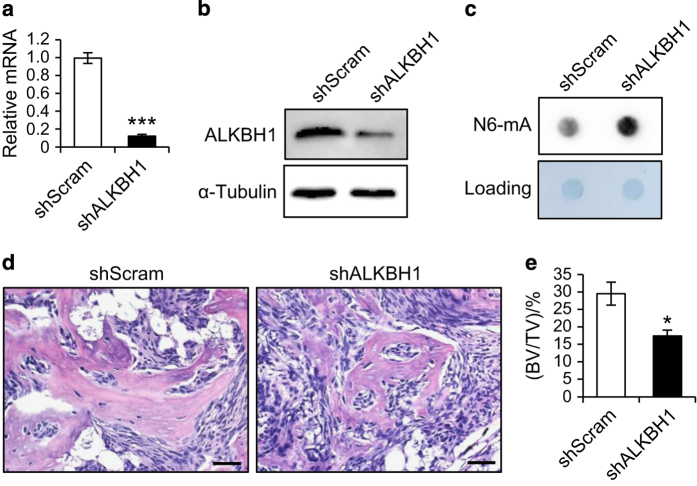
Depletion of ALKBH1 inhibits bone formation *in vivo*. (**a**) Real-time RT-PCR*. n*=3. ***P<0.001. (**b**) Western blot analysis. (**c**) Representative images of dot blot assay. (**d**) Hematoxylin and eosin staining of the ectopic bone formation. (**e**) Quantitative analyses of bone volume versus total tissue volume (BV/TV). *n*=5. *P<0.05.

To verify our *in vitro* findings, we examined whether the knockdown of ALKBH1 affected MSC-mediated bone formation *in vivo*. To this end, we generated the stable knockdown MSCs using lentiviruses expressing shRNA and implanted them with β-TCP carriers into immunocompromised mice subcutaneously. RT-PCR and western blot analysis showed that >85% of the ALKBH1 was depleted in MSCs expressing ALKBH1 shRNA (shALKBH1) compared with those expressing scrambled shRNA (shScram). The N6-mA levels in whole genomic DNA were increased. Notably, hematoxylin and eosin staining showed that ALKBH1-depleted cells formed less bone tissues (Figure 3d) than did the shScram cells. Quantitative measurement of mineralized tissue areas revealed a >40% decrease in bone formation (Figure 3e).

### Overexpression of ALKBH1 enhances osteoblastic differentiation of MSCs

**Figure 4:**
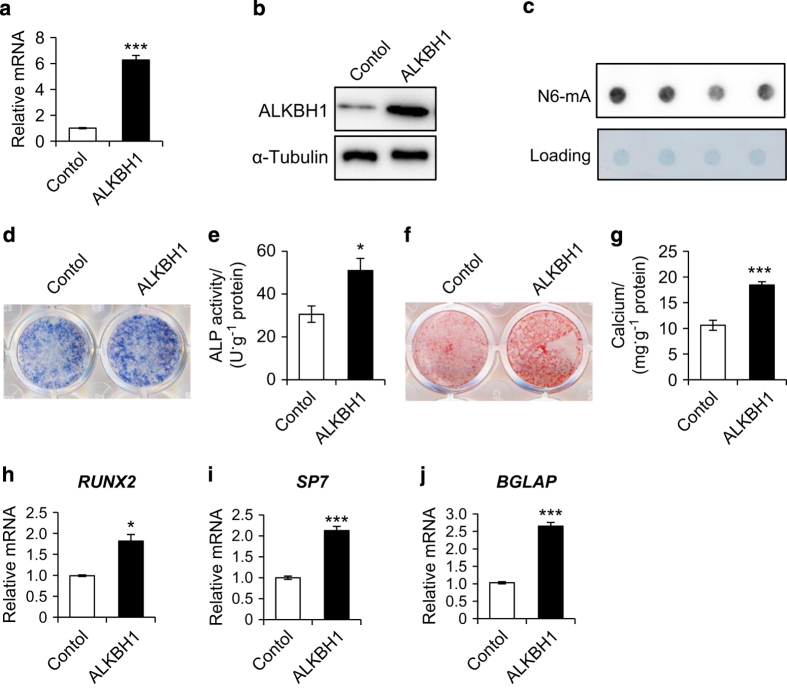
Overexpression of ALKBH1 enhances osteogenic differentiation. (**a**) Real-time RT-PCR*. n*=3. ***P<0.001. (**b**) Western blot analysis. (**c**) Representative images of dot blot assay. (**d**) Representative image of ALP staining. (**e**) Quantitative analyses of the ALP activity. *n*=5. *P<0.05. (**f**) Representative image of ARS staining of MSCs. (**g**) Quantitative analyses of calcium mineralization. *n*=5. ***P<0.001. (**h**–**j**) Real-time RT-PCR revealed increased messenger RNA expression of *RUNX2*, *SP7*, and *BGLAP*. *n*=3. *P<0.05 and ***P<0.001.

To investigate the effects of ectopic overexpression of ALKBH1on osteoblastic differentiation, human MSCs were stably transduced with lentiviruses expressing ALKBH1 (Figure 4a and b). As expected, ALKBH1 overexpression decreased the N6-mA levels in whole genomic DNA (Figure 4c). In addition, ALP activity and cell mineralization of MSCs were enhanced by the overexpression of ALKBH1 (Figure 4d–g). RT-PCR showed that the expression of osteogenic-related genes, such as *RUNX2*, *SP7*, and *GBLAP*, was significantly elevated after osteogenic induction for 7 days (Figure 4h–j).

### Depletion of ALKBH1 impairs ATF4 transcription

**Figure 5:**
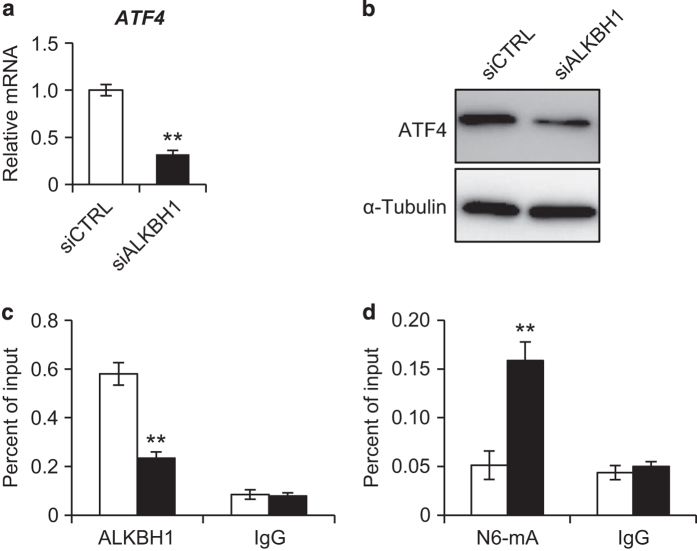
Depletion of ALKBH1 impairs ATF4 transcription. (**a**) Real-time RT-PCR showing decreased *ATF4* expression*. n*=3. **P<0.01. (**b**) Western blot analysis. (**c**) Chromatin immunoprecipitation (ChIP) assay for ALKBH1. ALKBH1 binds to the promoter region of *ATF4*. *n*=4. **P<0.01. (**d**) ChIP assay for N6-mA. Knockdown of ALKBH1 increases N6-mA levels on the promoter region of *ATF4*. *n*=4. **P<0.01.

ATF4 is a transcription factor that has a pivotal role in osteogenesis along with RUNX2 and Osterix. Interestingly, we found that the depletion of ALKBH1 in MSCs significantly reduced the ATF4 messenger RNA and protein levels after osteogenic reduction for 7 days (Figure 5a and b). More importantly, chromatin immunoprecipitation assays demonstrated that ALKBH1 binds to the promoter region of *ATF4* (Figure 5c). Knockdown of ALKBH1 restricted this binding (Figure 5c) and increased the abundance of N6-mA on the promoter (Figure 5d), which led to transcription silencing. These findings indicated that ALKBH1 may regulate the osteoblastic differentiation of MSCs by removing the N6-mA modifications on *ATF4*.

### ATF4 overexpression rescues the phenotypes

**Figure 6:**
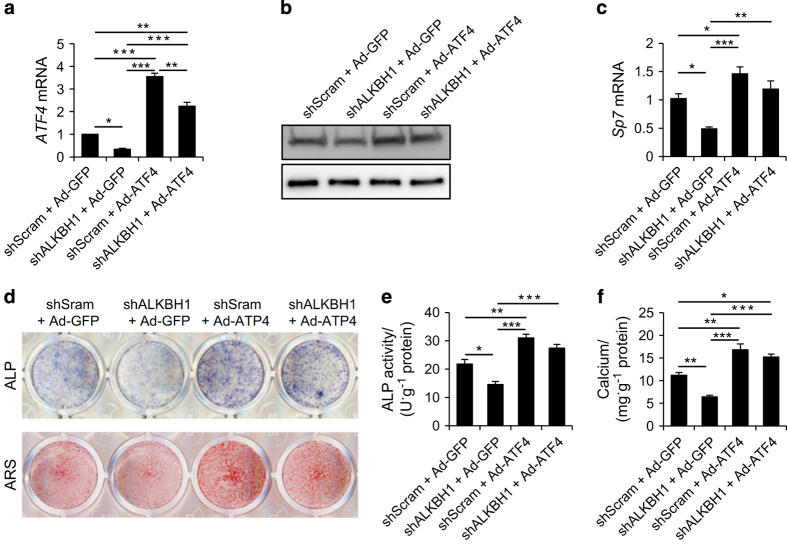
Overexpression of ATF4 rescues the phenotypes. (**a**) Real-time RT-PCR for *ATF4. n*=3. *P<0.05, **P<0.01 and ***P<0.001. (**b**) Western blot analysis for ATP4. (**c**) Real-time RT-PCR for *SP7*. *n*=3. *P<0.05, **P<0.01 and ***P<0.001. (**d**) Representative images of ALP staining and ARS staining. (**e**) Quantitative analyses of the ALP activity. *n*=5. *P<0.05, **P<0.01 and ***P<0.001. (**f**) Quantitative analyses of calcium mineralization. *n*=5. *P<0.05, **P<0.01 and ***P<0.001.

To further elucidate the mechanism, we performed rescue experiments by overexpressing ATF4 or control GFP in stable ALKBH1-depleted MSCs using adenoviruses. The successful transduction was confirmed by RT-PCR and western blot (Figure 6a and b). Ectopic ATF4 expression significantly increased the expression of *SP7*, a master transcription factor for osteogenic differentiation (Figure 6c). In addition, ALP activity and mineralization were rescued (shALKBh1+Ad-ATF4 vs shALKBh1+Ad-GFP; Figure 6d–f).

## Discussion

MSCs have garnered attention owing to their potential for osteogenic differentiation and regeneration therapy.^36–38^ Exploring the mechanism of MSC lineage specification and differentiation offers a brand-new perspective for clinical applications.^39^ In the present study, we found that the expression of ALKBH1 is upregulated during osteogenic differentiation *in vivo*. The depletion of ALKBH1 markedly increased the N6-mA levels and significantly reduced the expression of osteogenic-related genes, ALP activity, and ECM mineralization. By contrast, the ectopic overexpression of ALKBH1 enhanced the osteoblastic differentiation of MSCs. Mechanically, we found that ALKBH1 may regulate osteoblastic differentiation by removing N6-mA modifications on *ATF4*.

Previous studies have shown that ALKBH1, which was identified as a DNA demethylase for N6-mA in Embryonic stem cells, has a crucial function in early development by regulating genes that are involved in differentiation and pluripotency.^25,26^ In our study, ALKBH1 depletion inhibits bone formation both *in vivo* and *in vitro*. We further noticed an increase in N6-mA and reduction in osteogenic-related genes and indexes. Ougland *et al.* reported that ALKBH1 interacts with several core transcriptional factors, such as OCT4, SOX2, and NANOG, to maintain the pluripotency of Embryonic stem cell.^25,40,41^ Moreover, ALKBH1 may regulate microRNAs that are associated with the differentiation of neuronal cells.^21^ In contrast, mice lacking ALKBH1 display defects of small or missing eyes, especially in the right eye, and multiple defects in the craniofacial, sternum, and limb skeleton.^26^ Together with the findings on ALKBH1 by Nordstrand *et al.*, these data indicate that *Alkbh1* ^*−/−*^ mice exhibited an incomplete condensation of mesenchymal cells during ossification, which is consistent with our hypothesis.

Recently, ALKBH1 was discovered as a demethylase for DNA N6-mA, thus offering a new perspective for DNA methylation. However, there is wide acceptance that the DNA methylation always occurs on the C5 position of cytosine residues in CpG sites in DNA.^20,42^ Fu *et al.* ^22^ demonstrated that epigenetic activation of bone-specific genes mediated by promoter demethylation typically occurs when MSCs differentiate into osteoblasts. Moreover, Dansranjavin *et al.* ^23^ suggested that the inhibition of stem-cell-specific genes by promoter methylation is a crucial epigenetic mechanism during stem cell differentiation. In previous studies, Wu *et al.* demonstrated that an increase of N6-mA in Alkbh1^*−/−*^ cells leads to gene silencing and that most of these genes are developmental factors and lineage-specifying genes.^11^ Intriguingly, these genes are most markedly enriched on the X chromosome and Chr13, indicating that the increase in N6-mA inhibits the transcription on X chromosome, especially on young full-length LINE-1 transposons (L1 elements).^11^ Taken together, these data indicate that accumulation of N6-mA at L1 elements is related to the inhibition of nearby gene. Thus, N6-mA modifications have a great influence on the activation of differentiation genes. It would be interesting to explore the relationship between ALKBH1 and N6-mA, and the mechanisms that affect osteogenic differentiation and bone formation. Our result indicated an inverse correlation between ALKBH1 and N6-mA. In addition, the depletion of ALKBH1 *in vivo* leads to less bone tissue and decreased bone formation. However, fewer papers on DNA demethylases have been published compared with RNA demethylases, which needs further exploration.

In this study, we demonstrated that ALKBH1 binds to the promoter region of *ATF4.* The lack of ALKBH1 restricted this binding and increased N6-mA in this region, which led to transcription silencing. Our outcome suggested that ALKBH1 removes the N6-mA on ATF4 to regulate the osteogenic differentiation of human MSCs. ATF4, an osteoblast-enriched transcriptional factor of the CREB family, is indispensable for the latest phases of osteogenic differentiation,^43^ bone formation, and mineralization of the ECM.^44^ Previous studies have demonstrated that ATF4 promotes differentiation by upregulating the expression of osteoblast-specific genes, such as *RANKL*, and by promoting the synthesis of type I collagen, which is a main component of the ECM.^5,44^ These two distinct mechanisms are both dependent on the phosphorylation by RSK2.^45^ Taken together, these data suggested that ALKBH1 enhances osteogenic differentiation by interacting with *ATF4*.

It needs to be noted that our findings are based on the *in vitro* experiments. Further *in vivo* studies are expected. Given that Alkbh1^*−/−*^ in mice leads to embryonic and postnatal lethality,^26^ a tissue-specific mouse model is desired to further elucidate the role of ALKBH1 and DNA N6-mA in regulating osteogenic differentiation.

Collectively, we demonstrated that ALKBH1 enhances osteogenic differentiation by removing the N6-mA modifications on *ATF4*. Our results indicate that N6-mA modifications area mechanism for epigenetic regulation of osteogenic differentiation.

## Figures and Tables

**Table 1:** Primers for quantitative RT-PCR and ChIP-qPCR

Gene	Primers
For RT-PCR	
*ALKBH1 F*	AGAAGCGACTAAACGGAGACC
*ALKBH1 R*	GGGAAAGGTGTGTAATGATCTGC
*RUNX2 F*	GCAAGGTTCAACGATCTGAG
*RUNX2 R*	GGAGGATTTGTGAAGACGGT
*SP7 F*	GAGGCAACTGGCTAGGTGG
*SP7 R*	CTGGATTAAGGGGAGCAAAGTC
*BGLAP F*	GGCGCTACCTGTATCAATGG
*BGLAP R*	GTGGTCAGCCAACTCGTCA
*ATF4 F*	ATGACCGAAATGAGCTTCCTG
*ATF4 R*	GCTGGAGAACCCATGAGGT
*GAPDH F*	TCATTGACCTCAACTACATG
*GAPDH R*	TCGCTCCTGGAAGATGGTGAT
For ChIP	
*ATF4 F*	TGGCAGCAACAGCAACCATTA
*ATF4 R*	TGAGCCTCAGTTTCCTCATTTGTA

Abbreviations: ChIP-qPCR, chromatin immunoprecipitation-quantitative PCR; RT-PCR, reverse transcription-PCR.
